# Jujuboside A prevents sleep loss-induced disturbance of hippocampal neuronal excitability and memory impairment in young APP/PS1 mice

**DOI:** 10.1038/s41598-019-41114-3

**Published:** 2019-03-14

**Authors:** Sidra Tabassum, Afzal Misrani, Bin-liang Tang, Jian Chen, Li Yang, Cheng Long

**Affiliations:** 1 School of Life Sciences, South China Normal University, Guangzhou, 510631 PR China; 2 School of Life Sciences, Guangzhou University, Guangzhou, 510006 PR China; 3 Institute of Brain Research and Rehabilitation, South China Normal University, Guangzhou, 510631 PR China

## Abstract

Sleep deprivation (SD) is the hallmark of modern society and may increase risk of Alzheimer’s disease (AD). However, it is unclear how SD facilitates early cognitive impairments observed in AD models, as the underlying molecular mechanism is largely unknown. Here, we aim to investigate SD-induced cellular and molecular alterations in hippocampus of young APP/PS1 mice and whether jujuboside A (JuA) treatment could negate these alterations. Our results reveal that although SD causes spatial memory impairments in both genotypes, SD decreases frequency and amplitude of mEPSCs and pCREB levels in WT, but increases frequency and amplitude of mEPSCs, NMDAR, GluR1, pCaMKII (β, α) and decreases CREB levels in APP/PS1 mice, implicating that SD may facilitate abnormalities in young APP/PS1 mice via enhancing neuronal excitability. Moreover, JuA suppresses SD-induced enhancement of mEPSCs and prevents memory impairment in APP/PS1 mice. Further, whole-cell puff experiment suggests that JuA may function to activate GABAergic inhibition to reduce SD-induced enhancement of excitatory synaptic transmission in APP/PS1 mice. The present study reveals that sleep loss induces spatial memory impairment in an AD mouse model by disrupting the excitatory signaling pathway, and JuA prevents this via GABAergic mechanism.

## Introduction

Alzheimer’s disease (AD) is a neurodegenerative disorder, characterized by progressive decline in cognition, whose enormous social and economic burden is expected to rise sharply in the next few decades^1^. The neurodegeneration observed in AD has been associated with synaptic dismantling and progressive decrease in neuronal activity^2^, especially disruption of synaptic plasticity in the hippocampus^3,4^. Although AD has been and continues to be studied extensively, there is still no effective therapy for the prevention or cure of the devastating memory impairment associated with this disease.

It has long been appreciated that sleep disturbances are prevalent in AD patients, which include nocturnal arousal, increased or decreased total sleep time, and reversal of the day/night sleep pattern^5^. In the same context, several studies demonstrate a link between disrupted sleep and memory dysfunction in AD mouse models^6^. However, the associations between sleep disturbances and AD also raise a question about the possible causal role for sleep impairment in AD. In essence, disrupted sleep may represent a risk factor for the disease. In support of this hypothesis, various reports show that both self-reported sleep problems^7,8^ and sleep fragmentation^9^ increase the risk of developing dementia; mainly AD. These findings suggested that disrupted sleep might potentially trigger early onset of AD.

In contrast, sleep itself is very important for reducing the burden of plasticity on neurons and for normalizing synaptic strength while restoring neuronal selectivity and the ability to learn, all of which enhance the consolidation and integration of memories^10^. Memory formation is strongly linked to long-term changes in synaptic strength. High neuronal activity activates *N*-methyl-D-aspartate receptors (NMDARs) on the postsynaptic membrane and induces Ca^2+^ influx, which leads to a long-lasting increase in synaptic efficacy. In turn, this results in calcium/calmodulin-dependent protein kinase II (CaMKII) activation, which plays a critical role in plasticity and is responsible for α-amino-3-hydroxy-5-methyl-4-isoxazolepropionic acid receptor (AMPAR) activation at synapses^11^. This is followed by activation of cyclic AMP response element binding protein (CREB), a very important transcription factor involved in many central nervous system (CNS) functions including neurogenesis, neuroprotection, circadian rhythms, synaptic plasticity and memory formation^12^. CREB is phosphorylated during memory processes by various protein kinases, mainly CaMKII^13^. Disruption of CREB signaling leads to cognitive deficits in AD^14^, while increasing CREB through CREB vector microinjection into the cornus ammonis 1 (CA1) of the hippocampus restores cognitive deficits in an AD mouse model^15^. However, whether and how sleep loss affects above excitatory molecular pathway remained largely unknown.

Jujuboside A (JuA), a herbal medicine extracted from the dried seed of the jujube (semen *Ziziphi spinosae*), has been widely used over many years as a sedative and hypnotic drug in China, Japan, Korea and other oriental countries. Experimental studies show that JuA significantly reduces spontaneous activity in mammals, increasing the speed of falling asleep and prolonging sleep time^16,17^. Previously, it has suggested a neuroprotective role against oxidative stress, inflammation and cognitive impairments in the dementia mouse model^18^. However, the role of JuA on sleep loss-induced neurological effects and their association with AD is not well documented.

**Figure 1:**
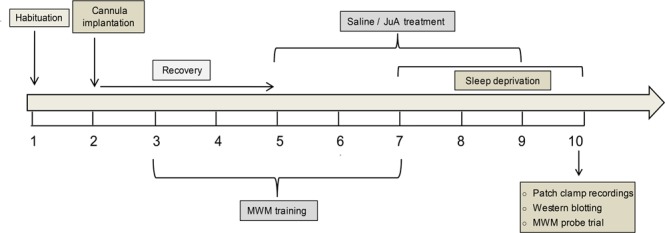
Schematic illustration of the experimental procedure. WT and APP/PS1 animals were randomly assigned to four groups (**A**) Ctrl, (**B**) SD, (**C**) JuA and (**D**) JuA + SD. Group C and D animals were given ICV injection of JuA (0.02 mg/kg) for five consecutive days starting two days before the SD procedure. Groups (**B**,**D**) animals were subjected to 72 h SD in individual automated treadmill cages. A set of mice from all groups was sacrificed for patch clamp recordings. Separate sets of animals were further subjected to either a spatial memory probe trial in the Morris water maze (MWM), or tissue collection for western blotting. Mice used for western blotting or electrophysiological experiments did not perform the behavioral test.

Utilizing 3–4 month-old APP/PS1 mice (young APP/PS1 mice) that don’t show any amyloid plaques or cognitive deficit at this age^19^ and wild-type (WT) littermates, we first investigated the molecular pathway sensitive to sleep loss and examined the influence of sleep loss on hippocampal electrophysiological properties of both genotypes. Moreover, we evaluated if sleep loss facilitates early cognitive impairment in young APP/PS1 mice. Second, we tested the hypothesis that JuA might prevent sleep loss-induced alterations in the AD mouse model (Fig. 1). Our study provides evidence that sleep loss enhances excitatory signaling activities and downregulates molecular marker of memory i.e. CREB in young APP/PS1 mice. Importantly, subchronic treatment with JuA prevents sleep loss-induced abnormalities in young APP/PS1 mice. To the best of our knowledge, this is the first study to provide electrophysiological, molecular, and behavioral evidence of the association between sleep loss, early cognitive impairment and neuroprotective role of JuA in an AD mouse model.

## Results

### Sleep loss induces an increase in NMDAR and GluR1 expression in young APP/PS1 mice

**Figure 2:**
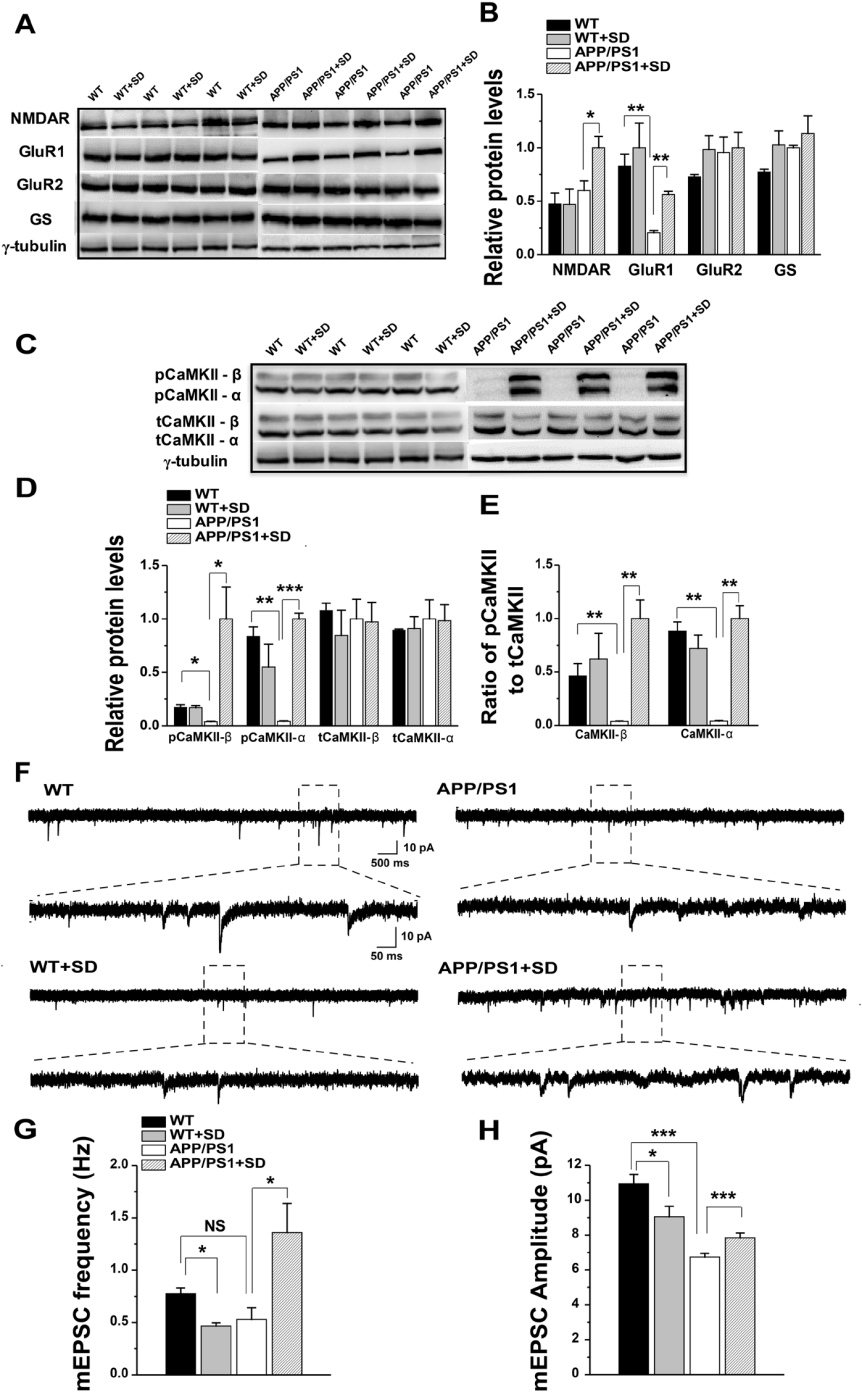
Sleep loss differentially affects miniature excitatory postsynaptic currents (mEPSCs) and excitatory signaling molecules of both genotypes. (**A)** Representative immunoblots of hippocampus extracts from WT, WT + SD, APP/PS1 and APP/PS1 + SD mice. **(B)** Quantification of immunoblots showing a significant increase in NMDAR and GluR1 levels in APP/PS1 + SD, while GluR2 and GS remain unchanged. **(C)** Representative immunoblots of β and α subunits of pCaMKII and tCaMKII from WT, WT + SD, APP/PS1, APP/PS1 + SD. **(D)** Quantification of immunoblots showing a significant increase in pCaMKII-β and α subunits, but no change in tCaMKII levels in APP/PS1 + SD mice. **(E)** Imbalance of pCaMKII/tCaMKII ratio between APP/PS1 and APP/PS1 + SD groups. **(F)** Sample traces showing mEPSCs recorded in the CA1 of the hippocampus in young WT, WT + SD, APP/PS1 and APP/PS1 + SD mice. **(G,H)** Quantification of mEPSCs reveals a significant decrease in both frequency and amplitude of WT (n = 11 cells/ 3 mice) and WT + SD (n = 14 cells/ 3 mice) groups and increase in frequency and amplitude of APP/PS1 (n = 14 cells/ 3 mice) and APP/PS1 + SD (n = 12 cells/ 3 mice) groups. Each value represents the mean ± SEM; *p < 0.05, **p < 0.01, ***p < 0.001; two-way ANOVA followed by LSD post hoc test.

To investigate sleep loss-induced molecular alterations, we performed western blotting for several key molecules. Glutamate is the most abundant excitatory neurotransmitter in brain; it binds to two types of postsynaptic glutamate receptors, NMDAR and AMPAR^20^, and has been shown to mediate AD pathogenesis^21^. Various reports show that the hippocampus is one of the earliest brain regions to display neurodegeneration and neurofibrillary tangles in AD^22^, and it plays a pivotal role in learning and memory^23^. To investigate whether sleep loss results in glutamatergic alterations in APP/PS1 mice, we measured the expression of various excitatory postsynaptic receptors in the hippocampus using western blotting. Analysis of NMDAR revealed no significant effect of genotype factor (F_(1,8)_ = 3.077, p = 0.117), but significant sleep factor (F_(1,8)_ = 8.506, p = 0.019) and insignificant genotype factor x sleep factor interaction (F_(1,8)_ = 3.203, p = 0.111). Post hoc analysis revealed that APP/PS1 + SD mice showed significant increase in NMDAR expression compared to APP/PS1 mice (p = 0.036; Fig. 2B). We also tested expression of two subtypes of AMPAR (GluR1, GluR2), and observed no significant effect of genotype factor (F_(1,8)_ = 4.155, p = 0.075), but significant sleep factor (F_(1,8)_ = 16.640, p = 0.003) and insignificant genotype factor x sleep factor interaction (F_(1,8)_ = 0.517, p = 0.492) in GluR1. Post hoc analysis revealed decrease in GluR1 expression in APP/PS1 as compared to WT (p = 0.009). Whereas, GluR2 analysis revealed no significant effect of genotype factor (F_(1,8)_ = 1.515, p = 0.25), sleep factor (F_(1,8)_ = 1.006, p = 0.345) and genotype factor x sleep factor interaction (F_(1,8)_ = 0.755, p = 0.410; Fig. 2B). To check whether these sleep loss-induced alterations in NMDAR and GluR1 levels was due to abnormal glutamate synthesis and metabolism, we measured expression levels of glutamine synthetase (GS), but found no significant genotype factor (F_(1,8)_ = 3.314, p = 0.106), sleep factor (F_(1,8)_ = 2.460, p = 0.155) and genotype factor x sleep factor interaction (F_(1,8)_ = 0.322, p = 0.585; Fig. 2B).

### Sleep loss induces an increase in levels of CaMKII in young APP/PS1 mice

It has been widely reported that CaMKII is activated by Ca^2+^ influx from NMDAR channels^24^ and it is also a potential substrate for driving AMPAR. We therefore aimed to determine the influence of increased NMDAR and GluR1 levels on the expression of CaMKII. We measured the levels of phosphorylated CaMKII (pCaMKII) β and α subunits. For pCaMKII-β our results revealed significant difference in genotype factor (F_(1,8)_ = 10.132, p = 0.012), sleep factor (F_(1,8)_ = 5.334, p = 0.049) and genotype factor x sleep factor interaction (F_(1,8)_ = 10.328, p = 0.012). Post hoc analysis revealed that APP/PS1 + SD mice showed significant increases in pCaMKII-β expression compared to APP/PS1 mice (p = 0.001). Moreover, western blot analysis of pCaMKII-α showed significant difference in genotype factor (F_(1,8)_ = 7.852, p = 0.023), but insignificant sleep factor (F_(1,8)_ = 2.054, p = 0.189) and statistically significant genotype factor x sleep factor interaction (F_(1,8)_ = 27.157, p = 0.00008). Whereas, post hoc analysis showed significant decrease in pCaMKII-α expression in APP/PS1 compared to WT mice (p = 0.001) and APP/PS1 + SD mice showed significant increase in pCaMKII-α expression compared to their age-matched non-SD APP/PS1 group (p = 0.0004; Fig. 2D). However, there was no significant statistical difference in levels of total CaMKII (tCaMKII)- β; genotype factor (F_(1,8)_ = 0.540, p = 0.483), sleep factor (F_(1,8)_ = 0.018, p = 0.894) and genotype factor x sleep factor interaction (F_(1,8)_ = 0.332, p = 0.580) and tCaMKII-α; genotype factor (F_(1,8)_ = 0.0001, p = 0.991), sleep factor (F_(1,8)_ = 0.468, p = 0.512) and genotype factor x sleep factor interaction (F_(1,8)_ = 0.015, p = 0.903). These results indicate a modification of the pCaMKII/tCaMKII ratio (Fig. 2E).

### Differential effects of sleep loss on hippocampal neuronal activity in WT and APP/PS1 mice

To explore the influence of sleep loss on hippocampal synaptic activities, we examined electrophysiological properties of hippocampal neurotransmission on young WT and APP/PS1 mice. Thus, we assessed whole-cell miniature excitatory postsynaptic currents (mEPSCs) in CA1 neurons of hippocampus in WT, WT + SD, APP/PS1 and APP/PS1 + SD and evaluated the frequency and amplitude of mEPSCs. Two-way ANOVA of mEPSC frequency revealed no significant effect of genotype factor (F_(1,47)_ = 3.103, p = 0.084), but significant sleep factor (F_(1,47)_ = 4.767, p = 0.034) and genotype factor x sleep factor interaction (F_(1,47)_ = 14.848, p = 0.0003). Moreover, mEPSC amplitude showed no significant effect of genotype factor (F_(1,47)_ = 0.797, p = 0.376), but significant sleep factor (F_(1,47)_ = 37.475, p = 1.760E-7) and genotype factor x sleep factor interaction (F_(1,47)_ = 11.375, p = 0.001). Our analysis showed a marked decrease in mEPSCs frequency and amplitude of WT + SD group as compare to WT control group animals (Fig. 2G,H), which is in support of downregulation of postsynaptic glutamine receptors and decrease in synaptic transmission following SD in normal rats^25,26^. However, post hoc analysis revealed significant increase in mEPSCs frequency and amplitude of APP/PS1 + SD mice as compared to non-SD APP/PS1 mice (Fig. 2G,H), implicating, likely, a neural excitotoxicity induced by sleep loss in the hippocampus of APP/PS1 mice.

### No change in levels of inhibitory postsynaptic receptors following sleep loss

**Figure 3:**
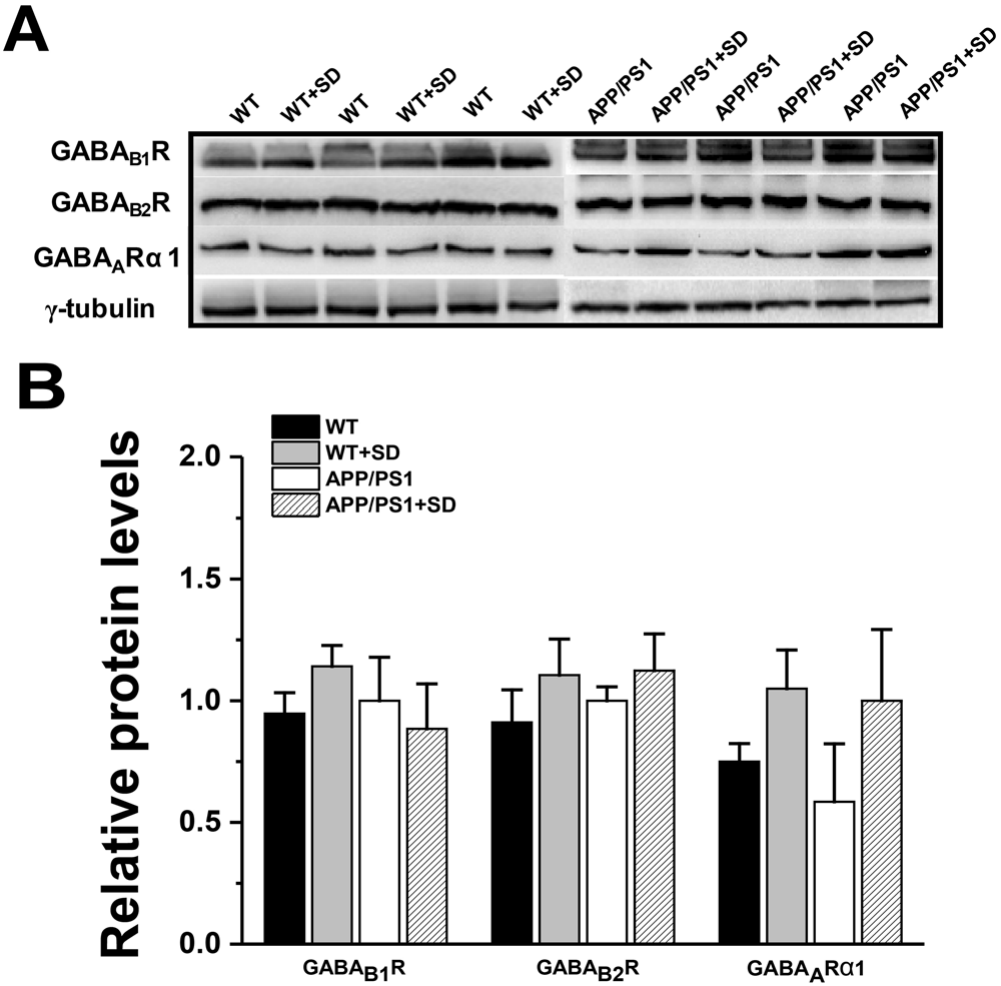
No change in inhibitory postsynaptic receptor expression among groups. (**A)** Representative immunoblots of hippocampus extracts from WT, WT + SD, APP/PS1 and APP/PS1 + SD mice. **(B)** Quantification reveals no significant difference in GABA_B1_R, GABA_B2_R and GABA_A_Rα1 expression among groups. Each value represents the mean ± SEM; two-way ANOVA followed by LSD post hoc test.

We next examined the levels of GABA receptors (GABARs) to further investigate the influence of sleep loss on inhibitory postsynaptic receptors in young APP/PS1 mice. In general, to avoid synaptic hyperexcitation, excitatory output of pyramidal neurons is precisely counterbalanced by input from inhibitory interneurons via binding of the presynaptically released neurotransmitter GABA to GABARs on the postsynaptic membrane^27^. However, when we measured the expression levels of three GABAR subunits, i.e. GABA_B1_, GABA_B2_ and GABA_A α1_, we found no significant difference in the levels of GABA_B1_; genotype factor (F_(1,8)_ = 0.076, p = 0.788), sleep factor (F_(1,8)_ = 0.514, p = 0.493) and genotype factor x sleep factor interaction (F_(1,8)_ = 1.187, p = 0.2307), GABA_B2_; genotype factor (F_(1,8)_ = 1.539, p = 0.249), sleep factor (F_(1,8)_ = 0.174, p = 0.686) and genotype factor x sleep factor interaction (F_(1,8)_ = 0.076, p = 0.788) and GABA_A α1_; genotype factor (F_(1,8)_ = 2.947, p = 0.124), sleep factor (F_(1,8)_ = 0.266, p = 0.619) and genotype factor x sleep factor interaction (F_(1,8)_ = 0.076, p = 0.788; Fig. 3A,B).

### JuA prevents sleep loss-induced increase in mEPSCs in young APP/PS1 mice

**Figure 4:**
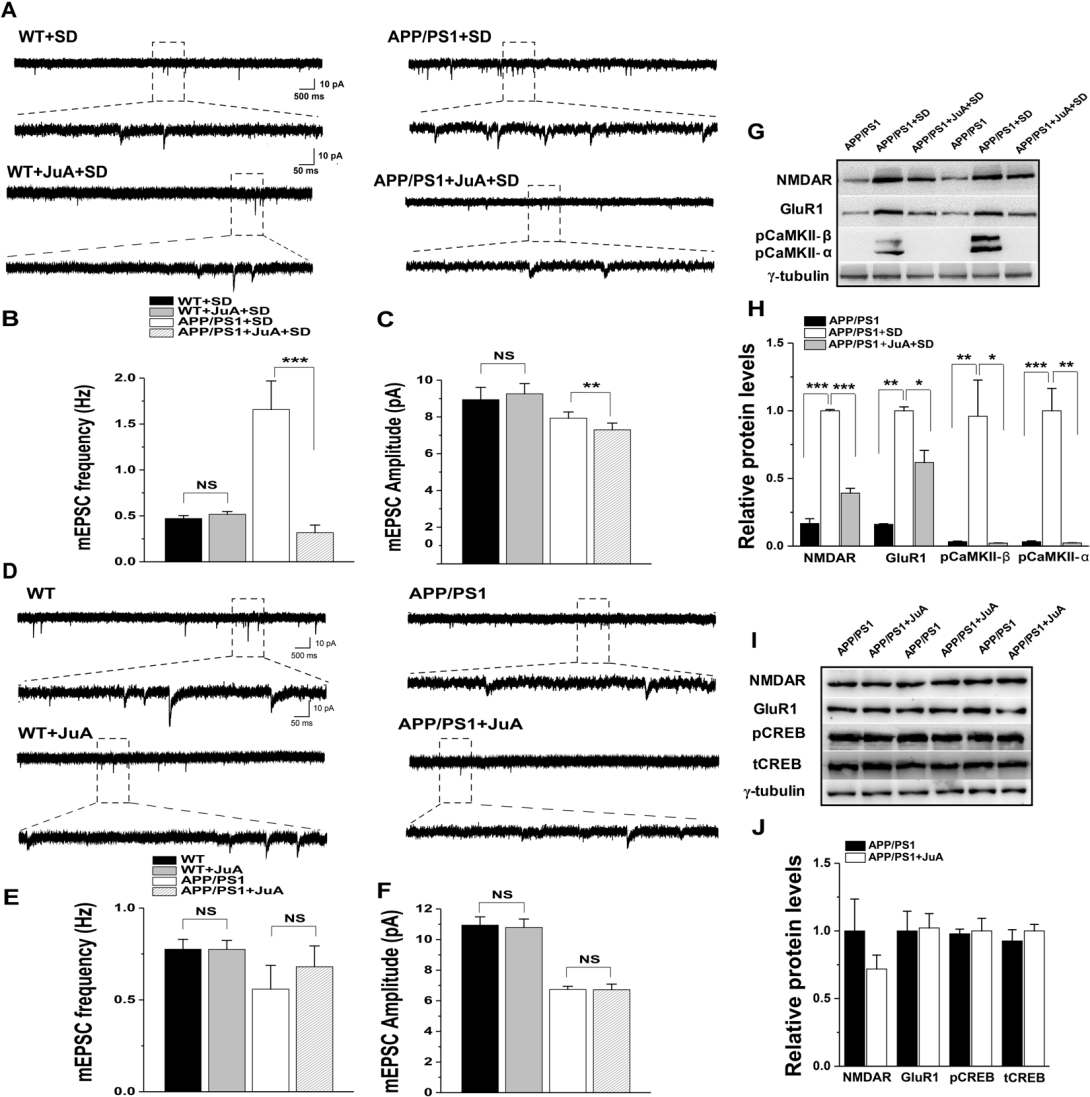
Treatment with JuA differentially affects sleep loss-induced alterations in WT and APP/PS1 mice. (**A)** Sample traces showing mEPSCs recorded in the CA1 of the hippocampus in WT + SD, WT + JuA + SD, APP/PS1 + SD and APP/PS1 + JuA + SD groups. **(B,C)** Quantification of mEPSCs reveals that JuA prevents sleep loss-induced increase in mEPSCs frequency and amplitude between APP/PS1 + SD (n = 10 cells/ 3 mice) and APP/PS1 + JuA + SD (n = 10 cells/ 3 mice) groups; while JuA treatment does not prevent sleep loss-induced decrease in mEPSCs frequency and amplitude in WT + SD (n = 14 cells/ 3 mice) and WT + JuA + SD (n = 15 cells/ 3 mice) group. **(D)** Sample traces showing mEPSCs recorded in the CA1 of the hippocampus in young WT, WT + JuA, APP/PS1 and APP/PS1 + JuA groups. **(E,F)** Quantification of mEPSCs amplitude and frequency shows no effect of JuA treatment in non-SD mice (n = 11 cells/ 3 mice for WT, n = 12 cells/ 3 mice for WT + JuA, n = 12 cells/ 3 mice for APP/PS1, n = 12 cells/ 3 mice for APP/PS1 + JuA). **(G)** Representative immunoblots of hippocampus extracts from APP/PS1, APP/PS1 + SD and APP/PS1 + JuA + SD mice. **(H)** Quantification of the immunoblots reveals that JuA treatment prevents sleep loss-induced increase in levels of NMDAR and GluR1 and pCaMKII- β and α in APP/PS1 mice. **(I)** Representative immunoblots of NMDAR, GluR1, pCREB and tCREB from APP/PS1 and APP/PS1 + JuA groups. **(J)** Quantification reveals no effect of JuA treatment on non-SD mice. Each value represents the mean ± SEM; *p < 0.05, **p < 0.01, ***p < 0.001; two-sample *t*-test, one-way ANOVA and two-way ANOVA analysis followed by Tukey’s and LSD post hoc test.

In this section, we tested our hypothesis that JuA treatment could prevent sleep loss-induced electrophysiological alterations in young mice. For this, we assessed mEPSCs in CA1 neurons of hippocampus in SD and JuA treated group animals using the whole-cell patch-clamp technique and evaluated the frequency and amplitude of mEPSCs. Data analysis of mEPSC frequency revealed significant sleep factor (F_(1,41)_ = 23.779, p = 1.667E-5), treatment factor (F_(1,41)_ = 13.787, p = 0.0006) and sleep factor x treatment factor interaction (F_(1,41)_ = 27.216, p = 5.568E-6; Fig. 4B). Importantly, post hoc analysis showed significant prevention by JuA of sleep loss-induced increase in the frequency and amplitude of mEPSCs in young APP/PS1 mice (p = 7.960E-8), however, JuA did not further reduce the frequency and amplitude of mEPSCs in of WT SD hippocampus (Fig. 4B,C). The mEPSC amplitude analysis revealed insignificant sleep factor (F_(1,39)_ = 0.081, p = 0.777), but significant treatment factor (F_(1,39)_ = 7.070, p = 0.011) and insignificant sleep factor x treatment factor interaction (F_(1,39)_ = 0.730, p = 0.39; Fig. 4C). The mEPSC frequency of WT and APP/PS1 mice treated with JuA revealed no significant genotype factor (F_(1,43)_ = 0.400, p = 0.530), treatment factor (F_(1,43)_ = 2.725, p = 0.106) and genotype factor x treatment factor interaction (F_(1,43)_ = 0.424, p = 0.518; Fig. 4E). Similarly, mEPSC amplitude analysis show insignificant genotype factor (F_(1,45)_ = 0.037, p = 0.847), but significant treatment factor (F_(1,45)_ = 96.407, p = 9.914E-13) and insignificant genotype factor x treatment factor interaction (F_(1,45)_ = 0.027, p = 0.869). It is worth noting that JuA only reduced the amplitude and frequency of hippocampal mEPSCs in APP/PS1 SD (Fig. 4B,C), but not in WT SD (Fig. 4B,C), WT and APP/PS1 mice (Fig. 4E,F), implicating a neural protective role of JuA possibly via suppressing SD-induced excitotoxicity of hippocampal CA1 in APP/PS1 mice.

### Amelioration of sleep loss-induced alterations in excitatory signaling proteins by JuA

Further, we tested the effects of JuA treatment on the sleep loss-induced alterations in excitatory pathway described above in young APP/PS1 mice. Data analysis revealed that sleep loss causes an increase in the expression levels of NMDAR (p = 5.732E-4), GluR1 (p = 0.003), pCaMKII-β (p = 0.012) and α (p = 4.065E-4; Fig. 4G,H). While five days of treatment with JuA not only maintained levels of NMDAR (p = 0.001) and GluR1 (p = 0.031) but also maintained levels of pCaMKII-β (p = 0.048) and α (p = 0.002) compared to APP/PS1 + SD mice (Fig. 4G,H). Furthermore, JuA treated non-SD APP/PS1 mice showed no significant difference in the expression levels of NMDAR, GluR1, phosphorylated CREB (pCREB) and total CREB (tCREB) (Fig. 4I,J).

### Sleep loss reduces basal levels of CREB, an effect counteracted by JuA

**Figure 5:**
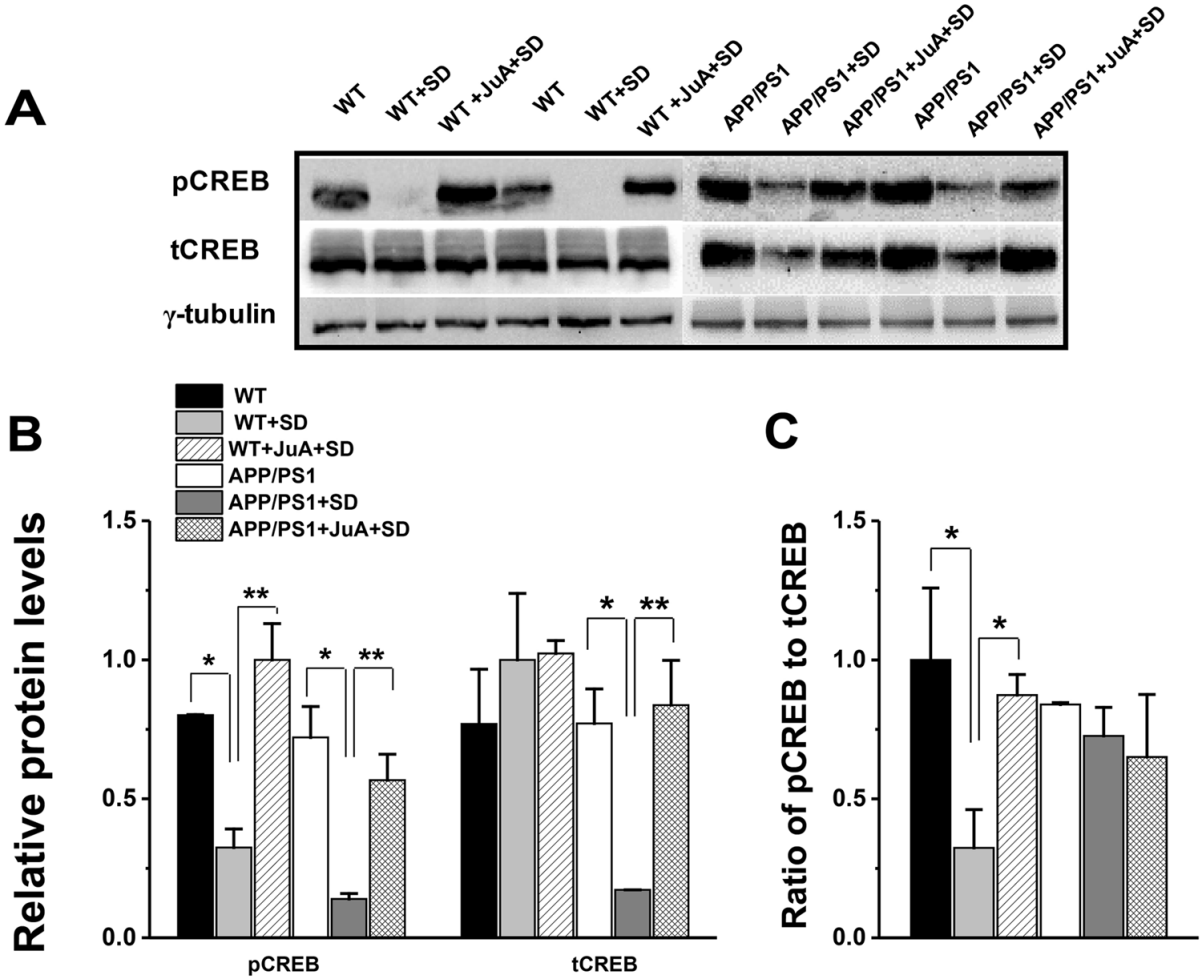
JuA maintains basal levels of CREB signaling. (**A)** Representative immunoblots of hippocampus extracts from WT, WT + SD, WT + JuA + SD, APP/PS1, APP/PS1 + SD and APP/PS1 + JuA + SD groups. **(B)** Quantification of the immunoblots showing significant decrease in pCREB levels in WT + SD and APP/PS1 + SD group and decrease tCREB levels in APP/PS1 + SD mice, while JuA treatment to sleep deprived WT and APP/PS1 mice maintains the levels of pCREB and tCREB **(C)** pCREB/tCREB ratio is unchanged for APP/PS1 but changed for WT. Each value represents the mean ± SEM; *p < 0.05, **p < 0.01; two-way ANOVA followed by LSD post hoc test.

To evaluate the outcome of sleep loss-induced molecular alterations, we measured pCREB and tCREB levels in the hippocampus of young mice, and investigated the effects of sleep loss and JuA on this pathway. CREB is downstream of CaMKII and is a major transcription factor involved in memory formation^28^. Previous studies have revealed a marked disruption in this pathway in an AD mouse model^29^. Our analysis for pCREB revealed significant sleep factor (F_(2,6)_ = 27.282, p = 9.722E-4) and treatment factor (F_(2,6)_ = 11.364, p = 0.015), but insignificant sleep factor x treatment factor interaction (F_(2,6)_ = 2.299, p = 0.181). Post hoc analysis showed significant difference in WT and WT + SD (p = 0.007) and APP/PS1 and APP/PS1 + SD groups (p = 0.002), whereas JuA treatment prevented SD-induced decrease in WT (p = 0.001) mice (Fig. 5A–C). However tCREB analysis revealed no significant sleep factor (F_(2,6)_ = 2.543, p = 0.158), but significant treatment factor (F_(2,6)_ = 7.325, p = 0.035) and insignificant sleep factor x treatment factor interaction (F_(2,6)_ = 4.067, p = 0.076; Fig. 5B). Post hoc analysis revealed significant difference in APP/PS1 and APP/PS1 + SD group (p = 0.03), whereas JuA treatment significantly prevented sleep loss-induced reduction in tCREB in APP/PS1 mice (p = 0.02; Fig. 5A–C).

### JuA ameliorates sleep loss-induced spatial memory deficit

**Figure 6:**
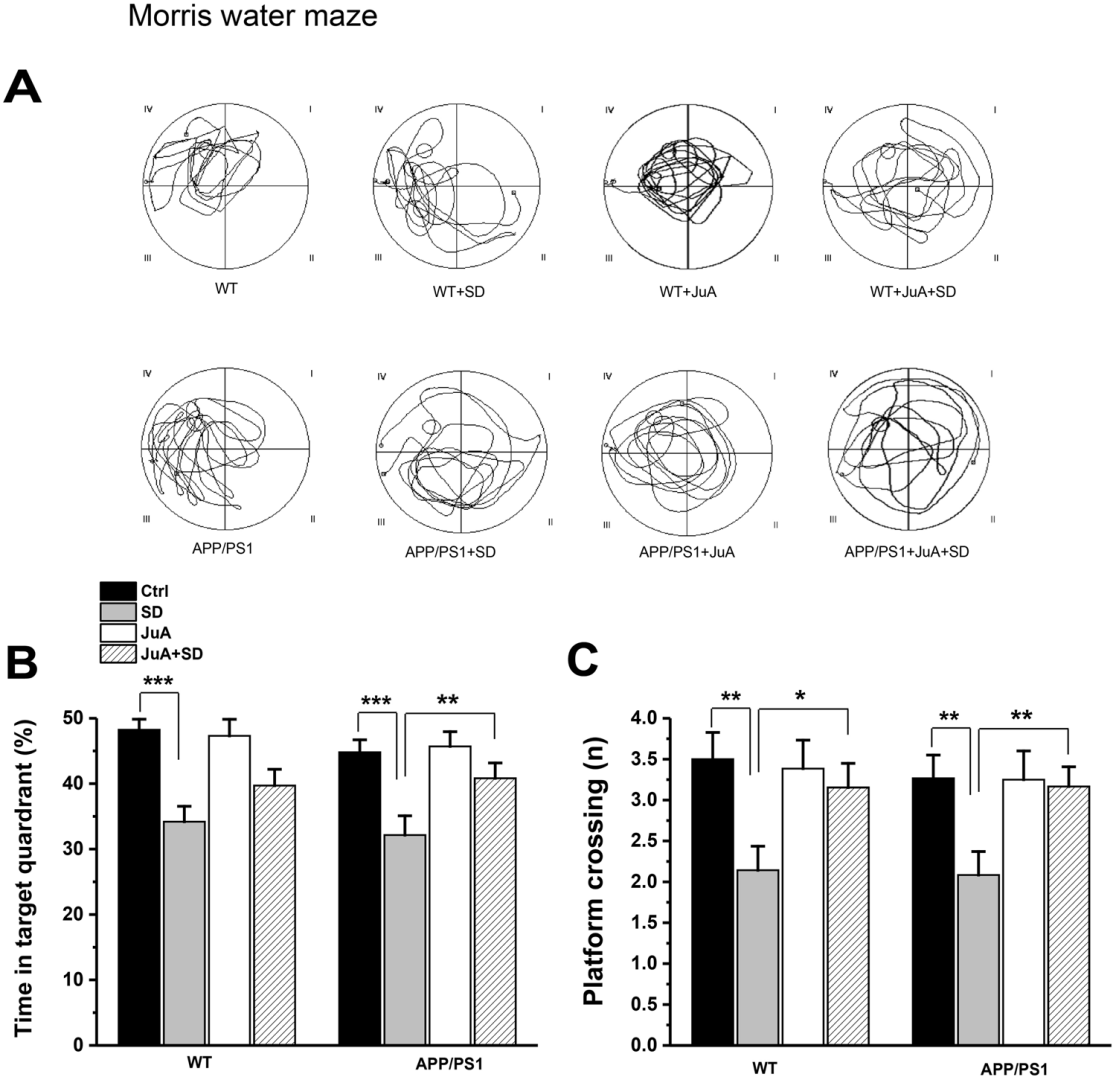
JuA prevents sleep loss-induced spatial memory deficit. (**A**) Sample traces of activity of mice in the MWM. **(B)** The WT + SD and APP/PS1 + SD spent less time in target quadrant, while APP/PS1 + JuA + SD group shows more time spent in the target quadrant. **(C)** Number of platform crossings of WT + JuA + SD and APP/PS1 + JuA + SD group differs from WT + SD and APP/PS1 + SD group respectively, but is comparable to their respective Ctrl animals **(**n = 14 for WT and WT + SD, n = 13 for WT + JuA and WT + JuA + SD, n = 15 for APP/PS1 and 12 for APP/PS1 + SD, APP/PS1 + JuA and APP/PS1 + JuA + SD). Each value represents the mean ± SEM; *p < 0.05, **p < 0.01, ***p < 0.001; two-way ANOVA followed by LSD post hoc test.

To explore the influence of sleep loss and JuA treatment on mouse behavior, we used the Morris water maze (MWM) to assess spatial memory in young WT and APP/PS1 mice. In WT mice, results demonstrate significant sleep factor (F_(1,50)_ = 22.818, p = 1.598E-5), but insignificant treatment factor (F_(1,50)_ = 1.019, p = 0.317) and sleep factor x treatment factor interaction (F_(1,50)_ = 2.027, p = 0.160). Post hoc analysis revealed significant deficit of spatial memory in the WT + SD group compared to non-SD WT mice (p = 4.529E-5). Furthermore, data analysis of APP/PS1 mice showed significant sleep factor (F_(1,47)_ = 13.812, p = 0.00053) and treatment factor (F_(1,47)_ = 4.127, p = 0.047), but insignificant sleep factor x treatment factor interaction (F_(1,47)_ = 2.719, p = 0.105). Post hoc analysis demonstrated significant deficit of spatial memory in the APP/PS1 + SD group compared to non-SD APP/PS1 mice (p = 3.0655E-4); as shown in Fig. 6B, sleep-deprived APP/PS1 mice took much longer time to find the hidden platform than the non-SD APP/PS1 mice, whereas JuA treatment significantly prevented sleep loss-induced spatial memory deficit in young APP/PS1 mice (p = 0.014). To further characterize this behavior, we analyzed another parameter i.e. the number of platform crossings, and data analysis of WT mice revealed significant sleep factor (F_(1,50)_ = 6.250, p = 0.015), but insignificant treatment factor (F_(1,50)_ = 1.988, p = 0.164) and sleep factor x treatment factor interaction (F_(1,50)_ = 3.144, p = 0.082). Post hoc analysis showed that WT + SD mice crossed the target platform fewer times than non-SD WT mice (p = 0.003), whereas JuA treatment significantly prevented sleep loss-induced spatial memory deficit in WT mice (p = 0.028, Fig. 6C). On the other hand, data analysis of APP/PS1 mice showed significant sleep factor (F_(1,47)_ = 4.600, p = 0.037), but insignificant treatment factor (F_(1,47)_ = 3.262, p = 0.077) and sleep factor x treatment factor interaction (F_(1,47)_ = 3.469, p = 0.068). Post hoc analysis demonstrates that APP/PS1 + SD mice crossed the target platform fewer times than non-SD APP/PS1 mice (p = 0.005), whereas JuA treatment significantly prevented sleep loss-induced spatial memory deficit in APP/PS1 mice (p = 0.014; Fig. 6C).

### JuA induces tonic GABA current-like response in hippocampal CA1 neurons

**Figure 7:**
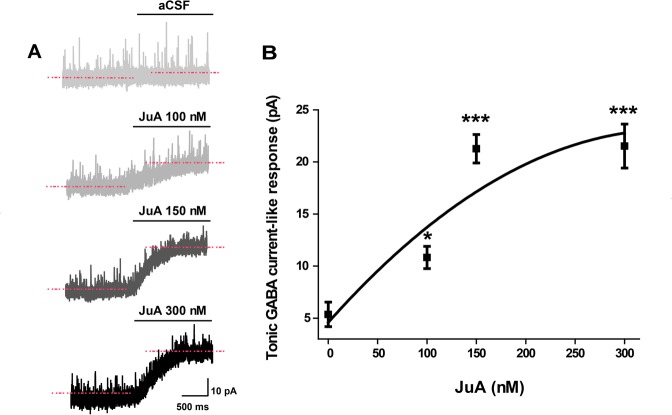
JuA induces tonic GABA current-like response. (**A**) Sample traces of tonic GABA current-like response in hippocampal slices from WT mice induced by aCSF and after addition of JuA (100, 150 and 300 nM) to aCSF; puff duration = 9 s, 4–6 psi. **(B)** Quantification of tonic GABA current amplitude shows a significant increase by JuA (aCSF, n = 12 cells; JuA 100 nM, n = 8 cells; JuA 150 nM, n = 10 cells; JuA 300 nM, n = 7 cells); error bars indicate SEM.

Finally, we evaluated the mechanism underlying the supression of JuA on sleep loss-induced enhancement of neuronal excitation. It is well established that GABAergic inhibitory mechanisms are crucial for the initiation and maintenance of sleep, most of the hypnotic drugs mediate GABARs activation to favor sleep^30^, and JuA has been speculated to work at GABA_A_Rs^31,32^. Our results reveal that puff application of JuA on hippocampal CA1 neuron evoked tonic GABA current-like response in a dose dependent manner (aCSF 2.18 ± 0.62; JuA 100 nM 12.20 ± 1.85, p = 0.039; JuA 150 nM 22 ± 3.36, p = 8.387E-9; JuA 300 nM 20.13 ± 4.89, p = 0.000; Fig. 7A,B), suggesting that JuA may improve sleep loss-induced behavioral and hippocampal molecular abnormalities via increasing GABAergic inhibition and thus maintaining proper excitation/inhibition balance.

## Discussion

Despite the prevalence of sleep disturbances in AD and the known effects of sleep loss on cognition, very little is known about the molecular mechanisms by which sleep disruption contributes to cognitive impairment in AD mouse model. An increasing number of people are suffering from sleep deficiency because of job-related burdens, social and economic stress, and sleep disorders. People with sleep deficiency have poor learning and memory abilities^33^, while recent studies on humans confirm that SD accelerates aging of the human brain^34^.

Although AD patients and mouse models of AD display anatomical changes within the hippocampus during the course of the disease, as well as deficits in the tasks that are dependent on normal functioning of this brain region. We argue that insufficient sleep may disrupt excitatory signaling pathways mainly in the hippocampus of young APP/PS1 mice that further leads to early cognitive decline. At first, to investigate molecular mechanism underlies sleep loss-induced alterations in young APP/PS1; we performed western blotting for several key molecules. Our western blotting result revealed increased NMDAR and GluR1 levels of APP/PS1 SD hippocampus when compared to non-SD APP/PS1 mice, whereas no significant difference was observed in WT mice when compared to WT SD mice (Fig. 2A,B). Though pCaMKII levels were significantly reduced in APP/PS1 when compared to that in WT mice, meanwhile, SD aberrantly increased pCaMKII levels (β and α) in APP/PS1 mice (Fig. 2C–E). These results suggest that SD might lead to excitotoxicity in the hippocampus of APP/PS1 mice.

Next, we carried out electrophysiological recordings in young WT and APP/PS1 mice, our results revealed that sleep loss differently affected hippocampal neuronal activities of WT and APP/PS1 mice. WT mice showed significant decrease in frequency and amplitude of mEPSCs after SD, which is consistent with the previous study demonstrating decrease in NMDA/AMPA ratio after 72 h SD^35^. Authors suggested that this reduction in NMDA/AMPA ratio is mainly because of reduction in excitatory currents induced by SD. Whereas, APP/PS1 mice exhibited significant increase in frequency and amplitude of mEPSCs following SD (Fig. 2F–H), though mEPSC amplitude of APP/PS1 mice was significantly reduced compared to that in WT mice indicating insufficient basal levels of postsyanptic excitatory response in 3~4 months APP/PS1 mice. As shown in a drosophila study where sleep loss interacts with Aβ to induce neuronal hyperexcitability^36^, sleep loss appeared to partially restore the amplitude of mEPSC in APP/PS1 mice. However, consider that soluble Aβ levels, though no Aβ plaques, are markedly increased in APP/PS1 mice at 3~5 months^37^. Various reports show that excessive activation of glutamate receptors can result in increased influx of Ca^2+^, leading to neuronal dysfunction, a process called excitotoxicity^38^, abnormal enhancing excitatory synaptic activity in APP/PS1 would lead to neural excitoxicity and thus facilitate AD pathogenesis^39,40^.

Importantly, JuA treatment prevented sleep loss-induced increase in the frequency and amplitude of mEPSCs in young APP/PS1 mice, but has no effect on sleep loss-induced mEPSC reduction of WT mice (Fig. 4A–C). It is previously reported that JuA has inhibitory effects on excitatory signaling pathway through anti-calmodulin action^16^, therefore, no effect of JuA on mEPSC of WT mice may implicate that JuA does not further decrease excitatory response when the latter reached low levels. As our results revealed that sleep loss in APP/PS1 mice caused enhancement in excitatory response and aberrantly increased levels of CaMKII compared to non-SD APP/PS1, but excitatory response and levels of CaMKII in WT SD mice remain unchanged compared to non-SD WT controls. Together, the data might explain why JuA changes sleep loss-induced mEPSCs abnormalities only in APP/PS1 mice but not in WT mice.

Western blotting results revealed that APP/PS1 SD mice treated with JuA exhibited similar levels of NMDAR, GluR1 and CaMKII compared to non-SD APP/PS1 mice (Fig. 4G,H). These findings are consistent with previous studies showing inhibitory effects of JuA on penicillin sodium-induced hyperactivity and glutamate-mediated excitatory signalling pathway^41,42^. Physiologically, NMDAR and AMPAR activation leads to channel opening at the plasma membrane and permeability to Ca^2+^. This rise in intracellular Ca^2+^ levels upon synaptic activity triggers the activation of several kinases, like CaMKII and CaMKIV^43^ which are upstream of CREB^12,44^ and critical for memory formation. We found a marked reduction in levels of not only pCREB but also of tCREB following SD in APP/PS1 mice (Fig. 5A–C), providing evidence that sleep loss-induced excitoxicity in young APP/PS1 mice further leads to a disruption of the activity of specific transcription factor i.e. CREB. In contrast, sleep loss only causes reduced pCREB levels in WT mice while tCREB remained unchanged (Fig. 5A–C). Previously, we and others have reported disruption of pCREB function following sleep loss in mice^45,46^, thus the present and previous studies suggest that WT and APP/PS1 animals may be affected differently when subjected to SD, as CREB is activated through various pathways^13^.

We also provide evidence that sleep loss impairs spatial memory in both young WT and APP/PS1 mice, as WT SD and APP/PS1 SD mice had difficulty to find the hidden platform in the MWM (Fig. 6A–C), which is consistent with previous studies showing worsening of memory following sleep loss in WT^45^ and AD mouse model^47^. CREB is a molecular marker of memory playing critical function in memory processing^48^, reduction in pCREB levels in both WT and APP/PS1 hippocampus after SD may contribute, at least in part, to memory impairment in both genotypes. Moreover, increased levels of pCREB ameliorated by JuA treatment plays an important role in preventing sleep loss-induced cognitive performance both in WT and APP/PS1 mice. Our study is in agreement with the previous study showing neuroprotective role of JuA against cognitive impairment in the dementia mouse model^18^.

It has been suggested that anxiety-like behavior is the early-stage behavioral biomarker of AD^49^. In our study, SD mice also exhibited increased anxiety-like behavior (Suppl Fig. 1), which is consistent with a previous report^50^. Moreover, this anxiety-like behavior was prevented by subchronic JuA treatment in APP/PS1 mice.

Sleep loss induces imbalance between glutamate and GABA receptors i.e. glutamate receptors significantly increased after sleep loss (Fig. 2A,B), whereas GABARs remained unchanged (Fig. 3A,B) in young APP/PS1 mice. Consider that the interregulation of the NMDAR and GABAR is crucial for homeostatic balance that regulates proper synaptic transmission; the tonic GABA current induced by JuA in hippocampal neurons (Fig. 7A,B) suggests that JuA may rebalance the excitation/inhibition through enhancing GABAergic inhibition in APP/PS1 SD mice. Hence, JuA treatment represents a promising means of counteracting sleep loss-induced alterations, and neuronal excitotoxicity in AD.

## Conclusions

**Figure 8:**
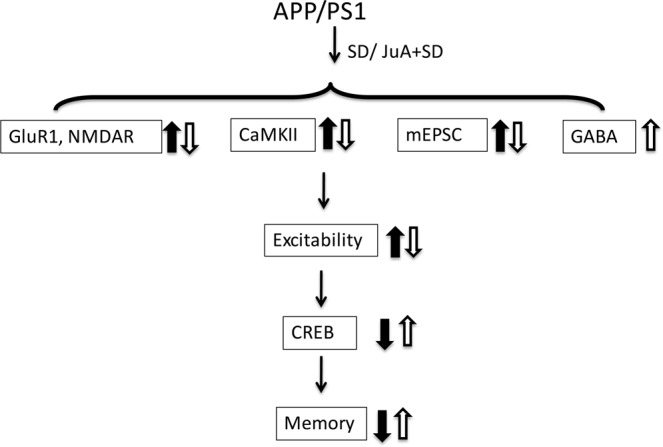
Graphical illustration of the principal pathway underlying SD-induced alterations in synaptic neurotransmission and cognitive decline: protection by JuA. Black arrow shows effects of SD while white arrow indicates effects of JuA on sleep loss-induced alterations in APP/PS1 mice.

Taken together, our study implies that sleep loss causes increase in excitatory signaling pathway that further reflected by increased frequency and amplitude of mEPSCs in APP/PS1 mice, implicating that SD might facilitate abnormalities in young APP/PS1 mice via enhancing neuronal excitability and sequential excitotoxicity. Conversely, WT mice exhibit significantly reduced mEPSC, suggesting that sleep loss differentially affects hippocampal neuronal activity in WT and APP/PS1 mice. Furthermore, our study provides evidence that JuA prevents sleep loss-induced disruption in excitatory signaling pathway and mEPSC in APP/PS1 mice by activation of tonic GABAergic inhibition (Fig. 8). Meanwhile, JuA treatment has beneficial effects on spatial memory and stabilization of CREB levels both in WT and APP/PS1 mice. The present study reveals that sleep loss induces a spatial memory impairment in AD, and JuA represents a promising target for the treatment of sleep loss-induced alterations in AD and other neurological disorders in which neural excitotoxicity is an issue.

## Materials and Methods

### Animal housing and study groups

Three to four-month-old female APP^swe^/PS1^ΔE9^ transgenic mice and their wild-type (WT) littermates from the animal house of the School of Life Sciences were used in this study. Mice were bred from a male heterozygote and a female WT mouse and were housed in a climate-controlled room (25 °C) under a 12 h light–dark cycle (lights on at 8:00 a.m.) with ad libitum access to standard rodent chow and water. All animal experiments performed in this study, were approved by the South China Normal University Animal Care and Use Committee, and were in accordance with the guidelines published in the National Institutes of Health Guide for Care and Use of Laboratory Animals.

Both WT and APP/PS1 animals were randomly assigned to the four different groups: Ctrl, SD, JuA and JuA + SD.

### Cannula implantation and treatment

Mice were positioned in a stereotaxic apparatus (RWD Life Science, Shenzhen, China) and a 26-gauge stainless steel guide cannula (catalog # 62102, RWD Life Science) was inserted into the right ventricle (AP, −0.2 mm, ML, +1.0 mm, DV, −3.0 mm) and fixed to the skull with dental cement. Erythromycin ointment was applied to the area around the cannula to avoid infection. All animal groups underwent the same surgical procedure. Mice were allowed to recover for three days before starting the daily intracerebroventricular (ICV) injection procedure. ICV microinjections were performed over a 30 s period and the injection needles remained in position for a further 30 s to facilitate diffusion of the drug.

JuA (purity ≥ 98%) was purchased from Chengdu Purechem-Standard Co. and dissolved in physiological saline solution (0.9% NaCl) at a stock concentration of 0.2 mg/ml. Because JuA is a macromolecular, polyphenolic compound that is predicted to have difficulty penetrating the blood–brain barrier^51^, it was administrated by ICV microinjection. JuA and JuA + SD group mice mice were given 0.02 mg/kg JuA for five consecutive days^18^, starting two days before the SD procedure, whereas Ctrl and SD mice were given equal volume of saline.

### Sleep deprivation

SD and JuA + SD group mice were sleep deprived for 72 h (Fig. 1) using the automated treadmill method as described previously^52^. Briefly, the treadmill system consisted of a plastic cage (*l*** × ***w* × *h* = 36 × 24 × 18 cm), which was fixed and suspended 0.1 cm above the vinyl belt of a treadmill, with the belt serving as the floor of the cage, and was automatically programmed to move slowly at a rate of 2 cm/s. To induce SD, the treadmill ran for 15 min and stopped for 60 min^53^; this SD protocol causes reduction of non-rapid eye movement (NREM) (20%) and rapid eye movement (REM) sleep (73%). Each mouse was singly housed in a treadmill cage; food and water were always available while the animals were on the treadmill. The timing of the light: dark cycle was the same as in the home cage. To habituate the mice to the treadmill environment, we programmed the treadmill (5 min on / 5 min off for 1 h) for two days prior to the SD procedure. WT and APP/PS1 group animals also obtained an equivalent amount of treadmill movement, but with a treadmill on/off schedule of 30 min on / 120 min off, allowing the animal longer periods of undisrupted sleep.

### Whole-cell patch-clamp

#### Slice preparation

Slices were prepared from all animal groups. SD and JuA + SD group animals were sacrificed immediately after SD, and slices prepared using a vibratome (VT 1000S, Leica, Germany) with ice–cold oxygenated cutting solution containing (in mM), 210 Sucrose, 3.0 KCl, 0.75 CaCl_2_, 3.0 MgSO_4_, 1.0 NaH_2_PO_4_, 26 NaHCO_3_, and 10 glucose (pH 7.2–7.4), saturated with 95% O_2_ and 5% CO_2_. Then 350 µm thick transverse hippocampal slices were cut using routine procedures^54,55^, kept in artificial cerebrospinal fluid (aCSF) containing (in mM) 124 NaCl, 3.0 KCl, 1.5 CaCl_2_, 1.3 MgSO_4_, 1.0 NaH_2_PO_4_, 26 NaHCO_3_ and 20 glucose (pH 7.2–7.4), and gassed with 95% O_2_/5% CO_2._ Slices were then allowed to recover for 90 min in a holding chamber at 30–32 °C before recording. Each slice was transferred to a submerged recording chamber with a PTCO_3_ proportional temperature controller and aCSF flow set to 2–3 ml/min.

#### Whole-cell patch-clamp recording in acute hippocampal slices

To record mEPSCs, 20 μM bicucullin and 1 μM tetrodotoxin (TTX) were added to aCSF^56^. The pipette was filled with the following internal solution (mM): 135 potassium gluconate, 10 KCl, 5 sodium phosphocreatine, 10 HEPES, 2 EGTA, 4 Mg·ATP and 0.5 Na_2_·GTP (pH 7.3, adjusted with KOH) at an osmolarity of 280–290 mOsm/L. To record tonic γ-aminobutyric acid (GABA) current-like response induced by puffing JuA, 10 mM 6-cyano-7-nitroquinoxaline-2,3-dione (CNQX) and 50 mM DL-2-amino-5-phosphonovaleric acid (APV) were added into the aCSF to block AMPA receptors and NMDA receptors respectively. The puff micropipettes were filled with JuA at 100 nM, 150 nM or 300 nM (dissolved in aCSF), and aCSF as vehicle control. Neurons were patched in voltage-clamp configuration and recorded at a holding potential of −60 or 0 mV separately. Data were collected with a MultiClamp 700 A amplifier and pCLAMP10 software (Molecular Devices, USA). The mEPSCs were analyzed by Mini Analysis Program (Synaptosoft Inc., USA) and were also checked visually.

### Western blotting

Western blotting was performed as described previously^56^. Briefly, mouse brains were homogenized with micro tissue grinders (Kimble) in 2 ml tubes, using lysis buffer containing 50 mM Tris pH 7.5, 150 mM NaCl, 5 mM EDTA, pH 8.0, 1% SDS, and protease inhibitor (Complete Mini; Roche). After 1 min homogenization, cellular debris was removed by centrifugation at 14000 × g for 10 min at 4 °C, and supernatant was collected for denaturation for 20 min at 75 °C. Tissue lysates were subjected to SDS-PAGE (Bio-Rad) and transferred to nitrocellulose membranes. The membranes were blocked for 30 min using 5% non-fat dry milk in Tris-buffered saline (TBS) containing 0.5% Tween-20 (TBST), then probed with specific primary antibodies against NMDAR1 (Abcam, ab109182), GluR1 (Abcam, ab31232), GluR2 (Abcam, ab206293**)**, GABA_B1_R (Abcam, ab55051), GABA_B2_R (Abcam, ab52248), GABA_A_Rα1 (Abcam, ab151573), GS (Abcam, ab73593), pCREB (Merck Millipore, clone 634-2), CREB (Abcam, ab178322), pCaMKII (Abcam, ab32678), and CaMKII (Abcam, ab52476) overnight at 4 °C. Anti γ-tubulin antibody (Sigma, T6557) was used as loading control. After three washes with TBST, HRP-labeled secondary antibody (CWS) was added at room temperature (RT) for 1 h using 5% milk in TBST followed by three additional washes with TBST. Bands were visualized using the Immobilon Western ECL system (CWS) and analyzed with Gel Pro Analysis software. For the presentation in figures, digitalized blot images were processed and cropped using Adobe Illustrator to show the interested protein signals. Full-length blots for the preparation of Figs 2(D,F), 3(A), 4(G,I), 5(A) are included as supplementary information. Blots in Supplementary Figs S2–S6 were cut into strips for probing with different primary antibodies.

### Morris water maze

The MWM is a widely accepted paradigm for testing spatial learning and memory in rodents. Mice were trained in the MWM before being subjected to SD (Fig. 1), in accordance with a protocol reported previously^57^. Briefly, mice were trained to find a hidden platform in a circular pool (51 cm deep and 122 cm in diameter) filled with water at 24–26 °C over four trials per day for five consecutive days. The platform was positioned in the same quadrant, but the starting point was changed in each of the four trials. The trial consisted of gently placing the animal in the water, facing the wall of the water tank, and allowing it to locate the hidden platform. Each trial continued until the mouse found the hidden platform, or for a period of 60 s maximum, after which the experimenter directed unsuccessful animals to the platform. The mouse was permitted to sit on the platform for 15 s. After the training period, a probe trial was conducted for 60 s in which the platform was removed from the targeted quadrant. Mouse activity in the above behavioral apparatus was collected by a digital video camera connected with a computer-controlled system (Zhenhua, Anhui, China). Tests were performed during the light period (8:00–11:00 am) by independent experimenters who were blind to the treatment schedule and genotype.

### Statistical analysis

Data were analyzed using OriginPro 2017 software. Statistical analysis was either Student’s *t*-test for two-group comparisons, one-way ANOVA and two-way ANOVA followed by Tukey’s or LSD post hoc for multiple group comparisons; unless otherwise stated, with p < 0.05 considered statistically significant. The data are presented as mean ± SEM.

## Supplementary information

Supplementary material

## Data Availability

The data generated in the present study are available from the corresponding author upon reasonable request.
