# Supplementary material for: Jujuboside A prevents sleep loss-induced disturbance of hippocampal neuronal excitability and memory impairment in young APP/PS1 mice
Source: Sci Rep. 2019 Mar 14;9:4512. doi: 10.1038/s41598-019-41114-3 (PMC6418242; doi:10.1038/s41598-019-41114-3)
Supplement: Supplementary file 1 — Supplementary material [file 41598_2019_41114_MOESM1_ESM.pdf]

Jujuboside A prevents sleep loss-induced hippocampal neuronal hyperexcitation and memory impairment in young APP/PS1 mice

Sidra Tabassum[1†], Afzal Misrani[1†], Bin-liang Tang[1], Jian Chen[1], Li Yang[2], Cheng Long[1,3*]

1 School of Life Sciences, South China Normal University, Guangzhou 510631, PR China

2 School of Life Sciences, Guangzhou University, Guangzhou 510006, PR China

3 Institute of Brain Research and Rehabilitation, South China Normal University, Guangzhou 510631, PR China

Supplementary material

S1

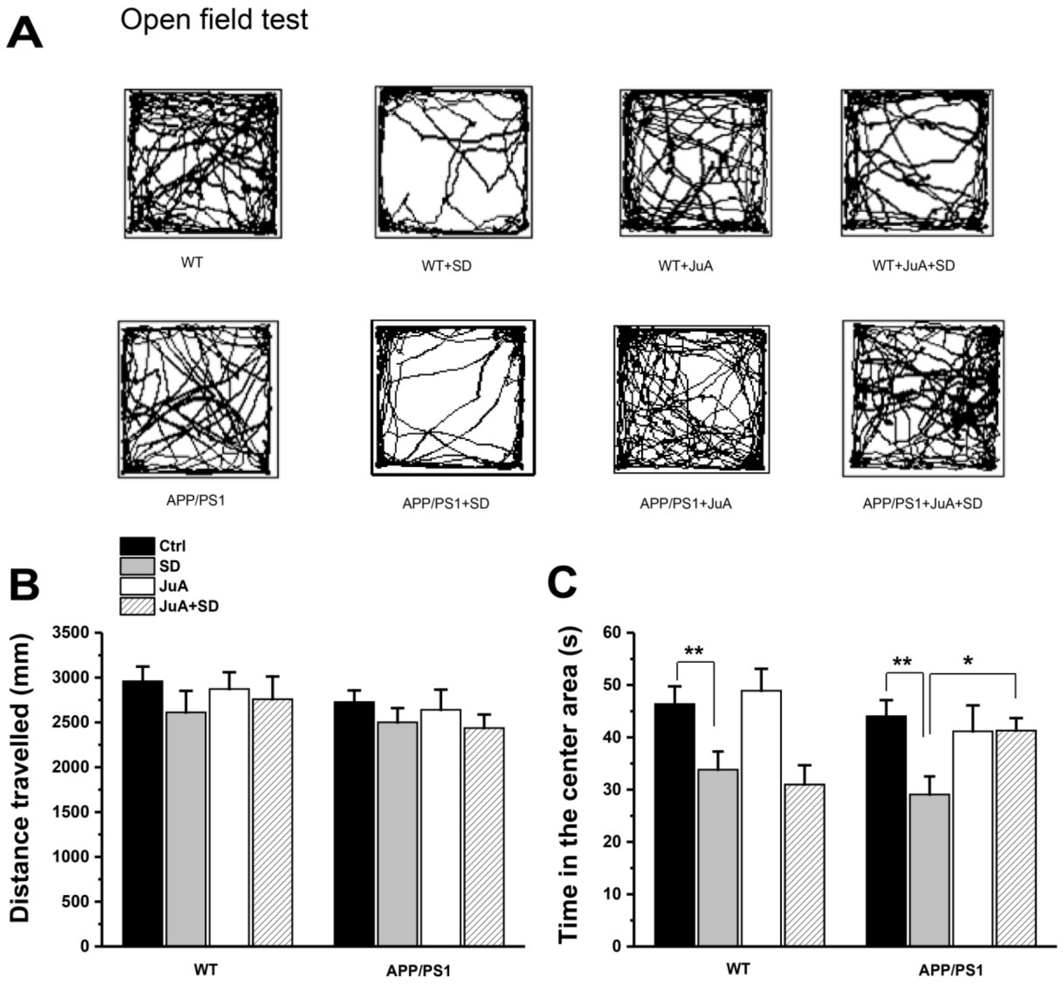

Supplementary figure 1 (S1): JuA prevents SD-induced anxiety-like behavior in young APP/PS1 mice. (A) Sample traces of locomotor activity in the OFT. (B) All groups show comparable locomotor activity. (C) Mice from the WT+SD and APP/PS1+SD group show a reduced time spent in the central area of the field, while JuA treatment shows anxiolytic activity in APP/PS1 mice by increasing the time spend in the central area (n = 12 for WT, n = 9 for WT+SD, WT+JuA and WT+JuA+SD, n = 16 for APP/PS1, 14 for APP/PS1+SD, APP/PS1+JuA+SD, n = 11 for APP/PS1+JuA). Each value represents the mean ± SEM; *p < 0.05, **p < 0.01; two-way ANOVA followed by LSD post hoc test.

S2.A

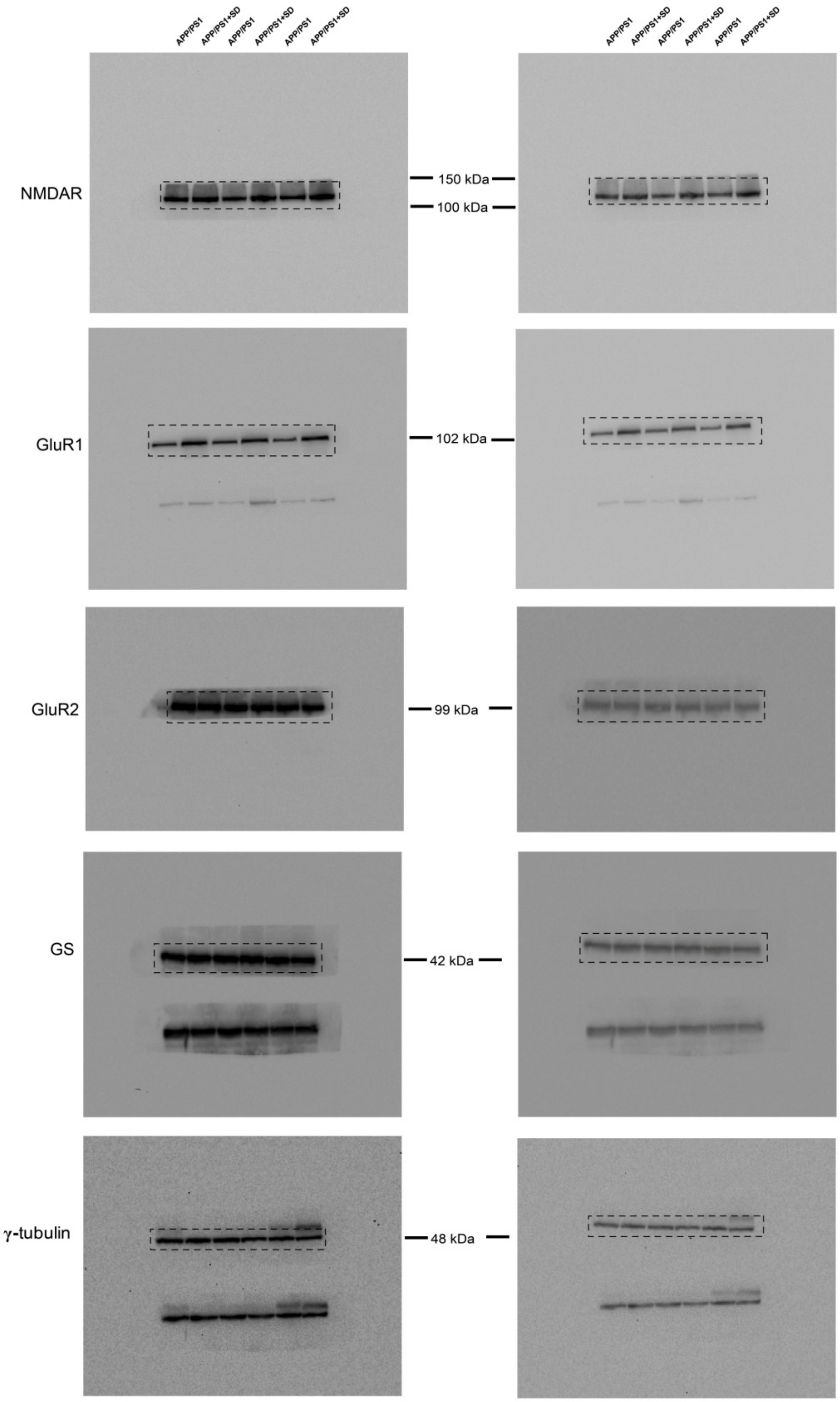

Supplementary figure 2.A (S2.A): Full length images of blots for Fig 2 D in main paper at two different exposures. Dotted rectangles indicate the regions used in the figures. Nitrocellulose membrane was cut before probing with the respective primary antibody.

S2.B

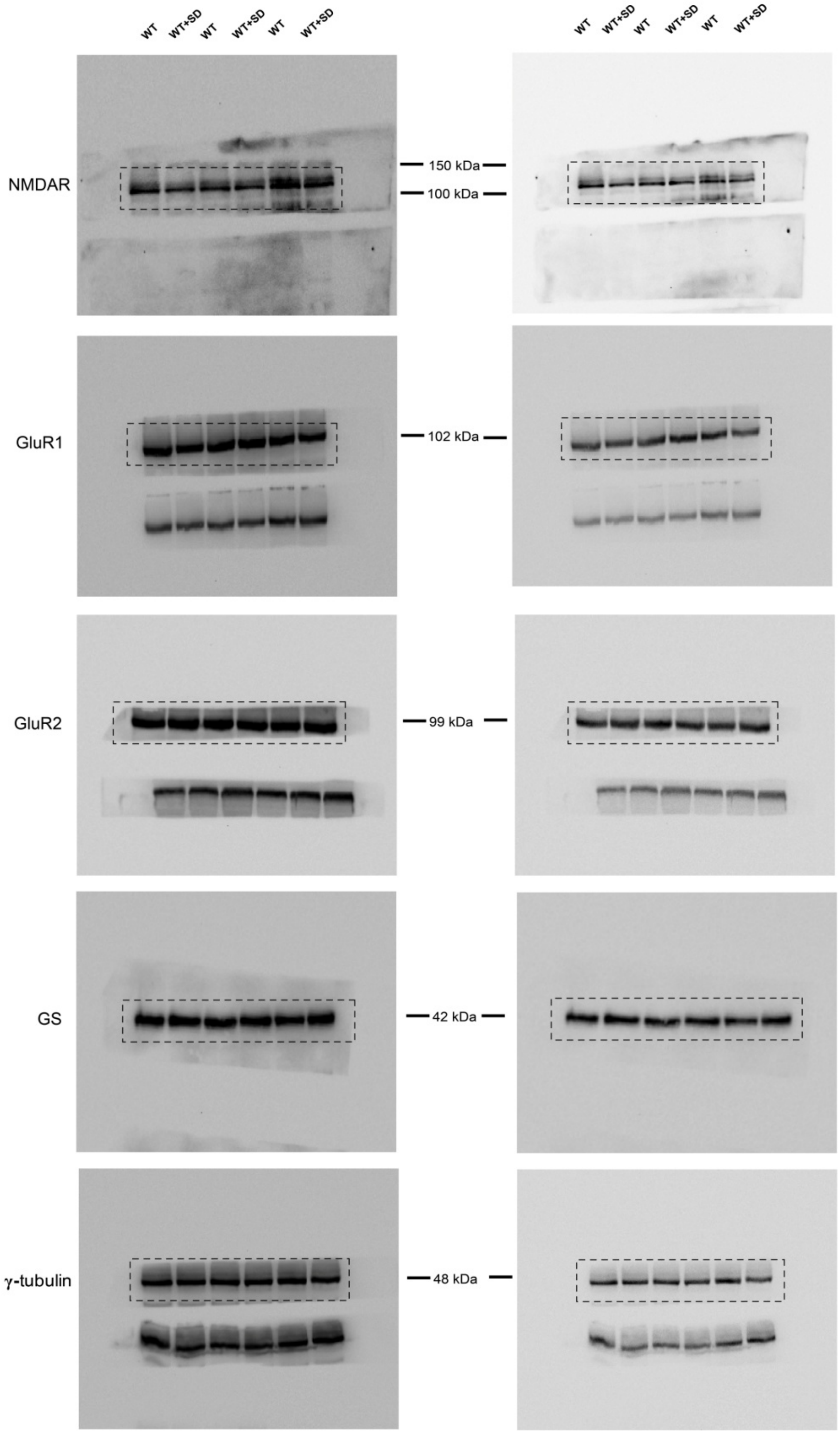

Supplementary figure 2.B (S2.B): Full length images of blots for Fig 2 D in main paper at two different exposures. Dotted rectangles indicate the regions used in the figures. Nitrocellulose membrane was cut before probing with the respective primary antibody.

S3.A

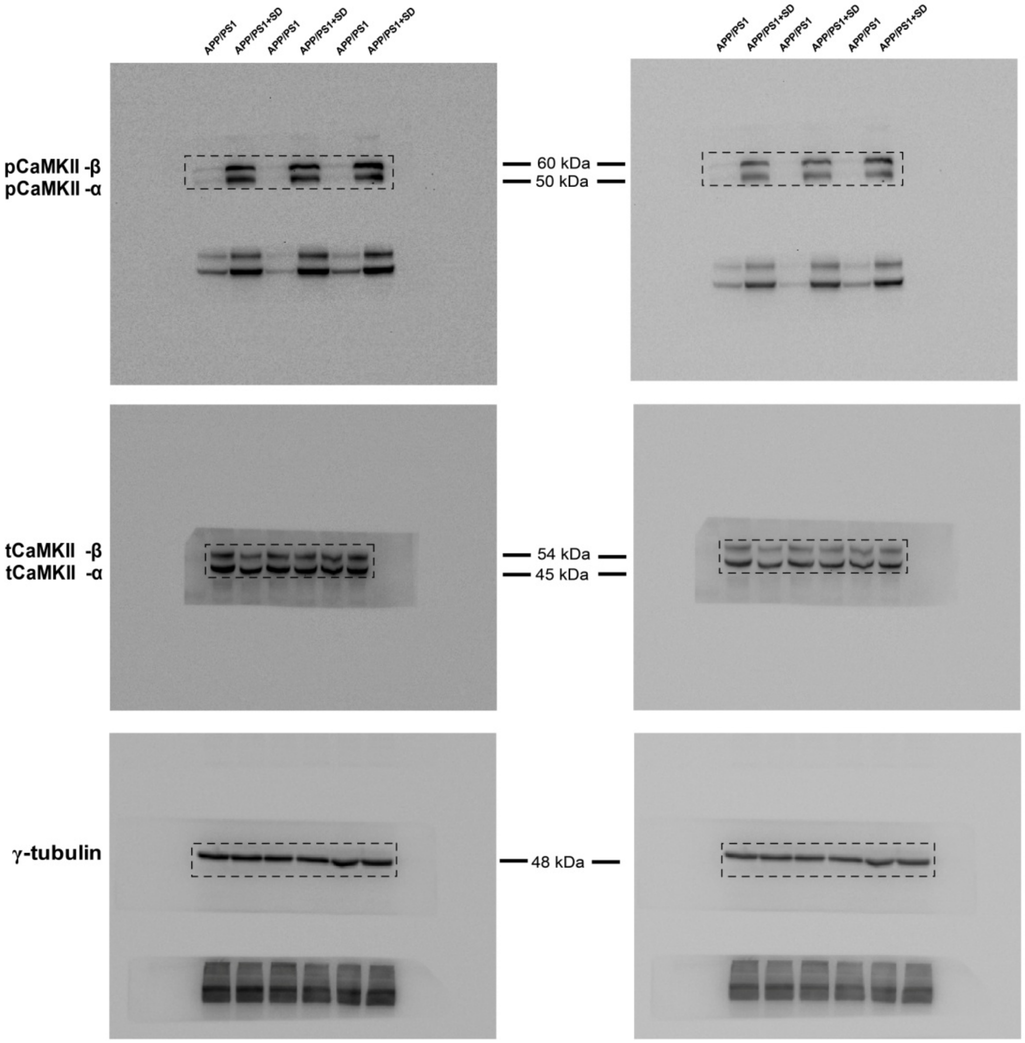

Supplementary figure 3.A (S3.A): Full length images of blots for Fig 2 F in main paper at two different exposures. Dotted rectangles indicate the regions used in the figures. Nitrocellulose membrane was cut before probing with the respective primary antibody.

S3.B

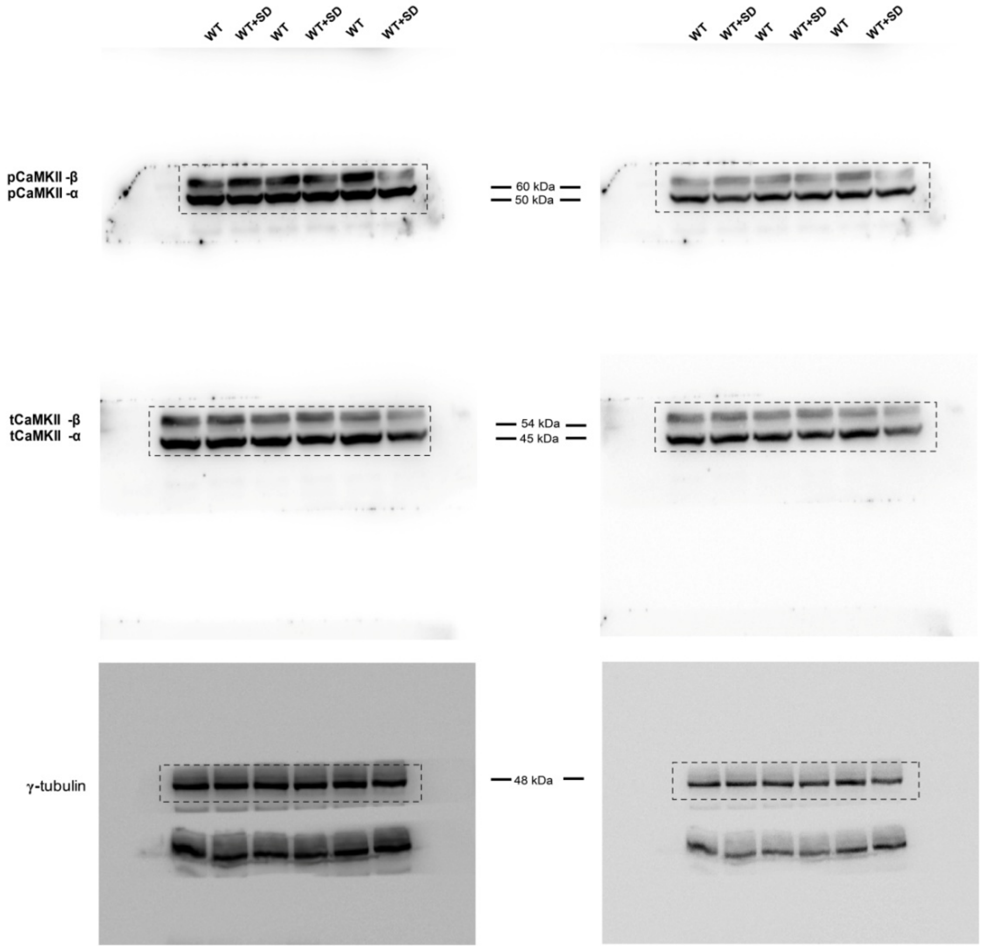

Supplementary figure 3.B (S3.B): Full length images of blots for Fig 2 F in main paper at two different exposures. Dotted rectangles indicate the regions used in the figures. Nitrocellulose membrane was cut before probing with the respective primary antibody.

S4.A

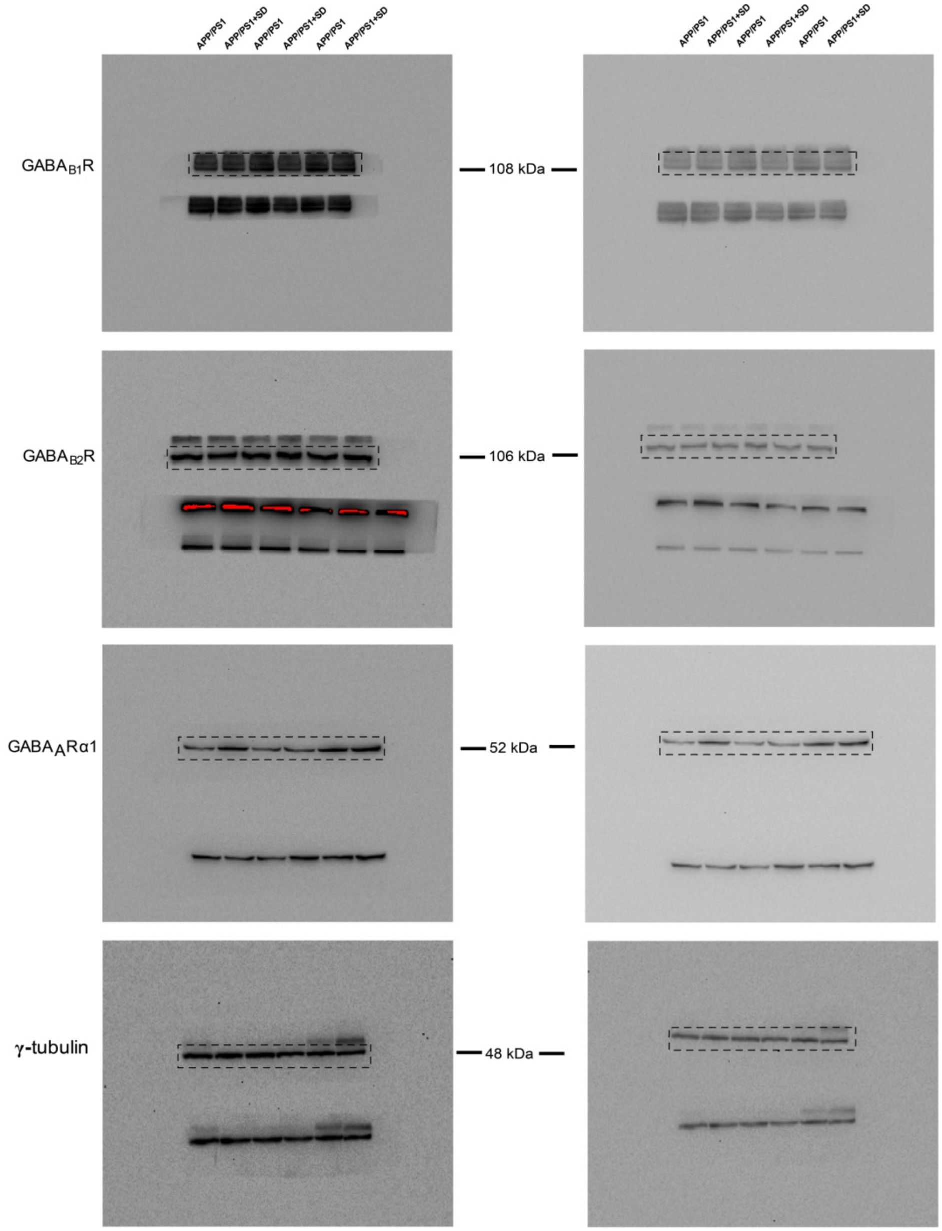

Supplementary figure 4.A (S4.A): Full length images of blots for Fig 3 A in main paper at two different exposures. Dotted rectangles indicate the regions used in the figures. Nitrocellulose membrane was cut before probing with the respective primary antibody.

S4.B

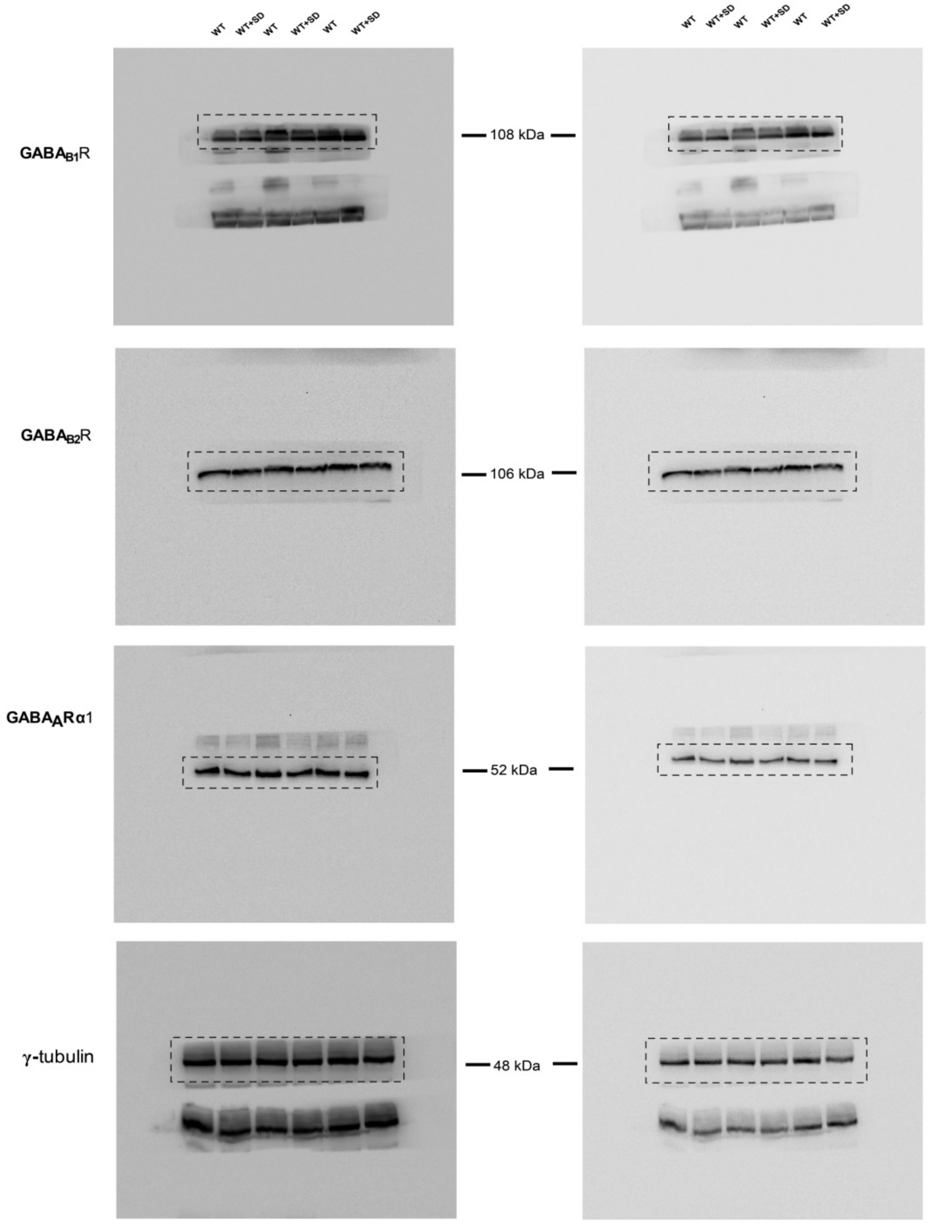

Supplementary figure 4.B (S4.B): Full length images of blots for Fig 3 A in main paper at two different exposures. Dotted rectangles indicate the regions used in the figures. Nitrocellulose membrane was cut before probing with the respective primary antibody.

S5.A

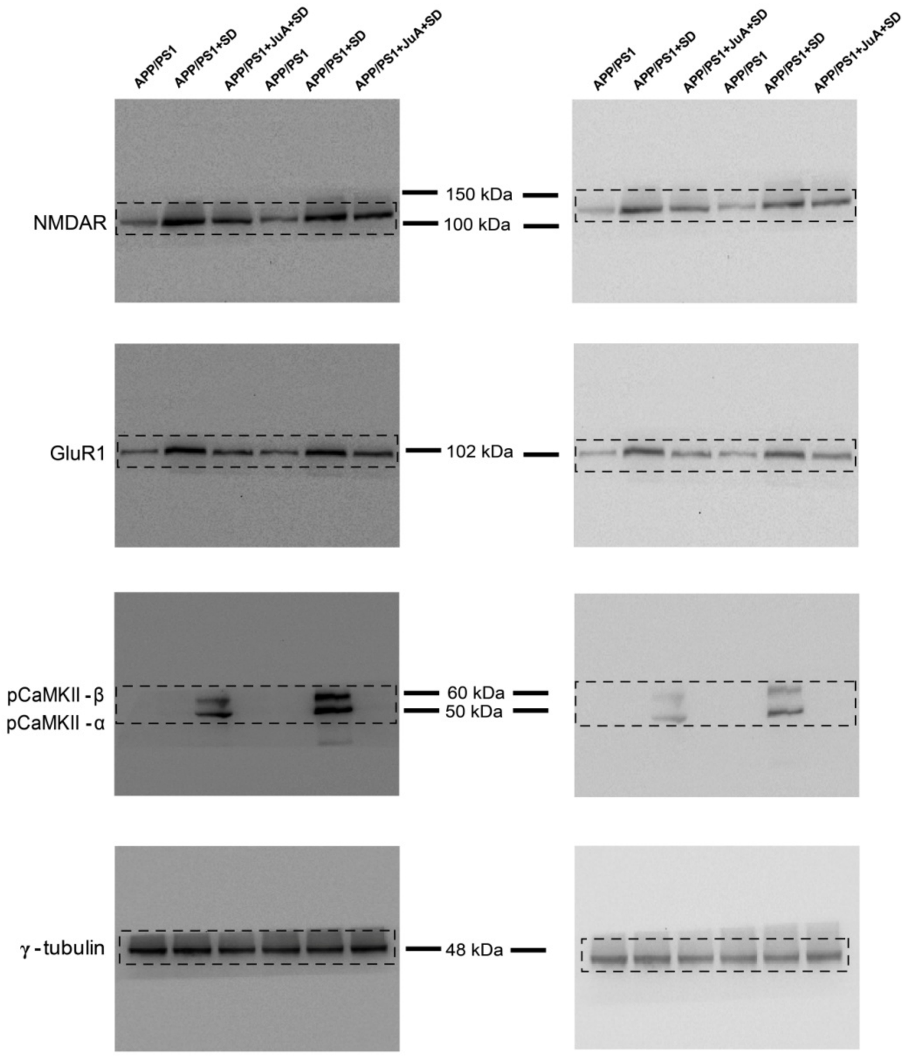

Supplementary figure 5.A (S5.A): Full length images of blots for Fig 4 G in main paper at two different exposures. Dotted rectangles indicate the regions used in the figures. Nitrocellulose membrane was cut before probing with the respective primary antibody.

S5.B

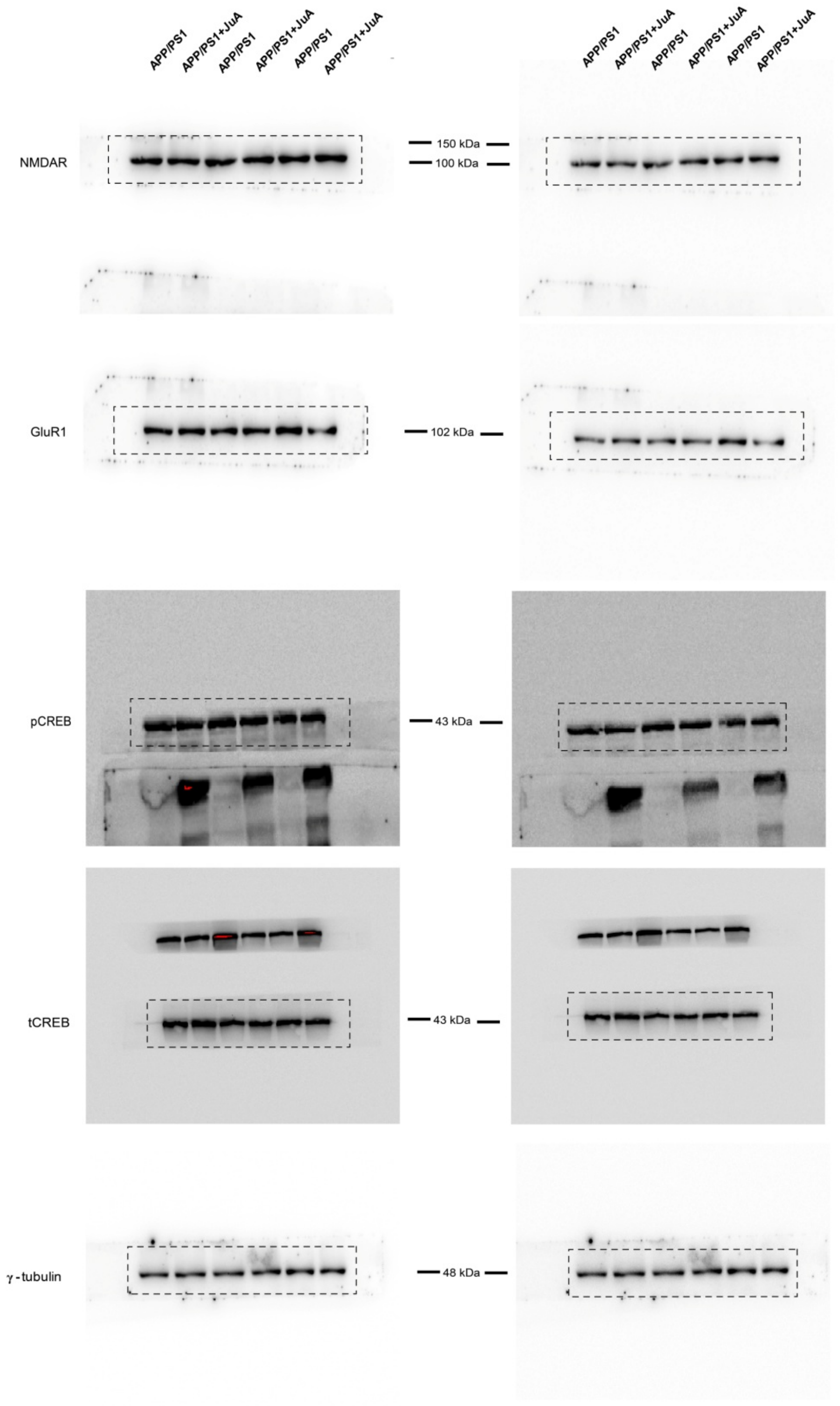

Supplementary figure 5.B (S5.B): Full length images of blots for Fig 4 I in main paper at two different exposures. Dotted rectangles indicate the regions used in the figures. Nitrocellulose membrane was cut before probing with the respective primary antibody.

S6.A

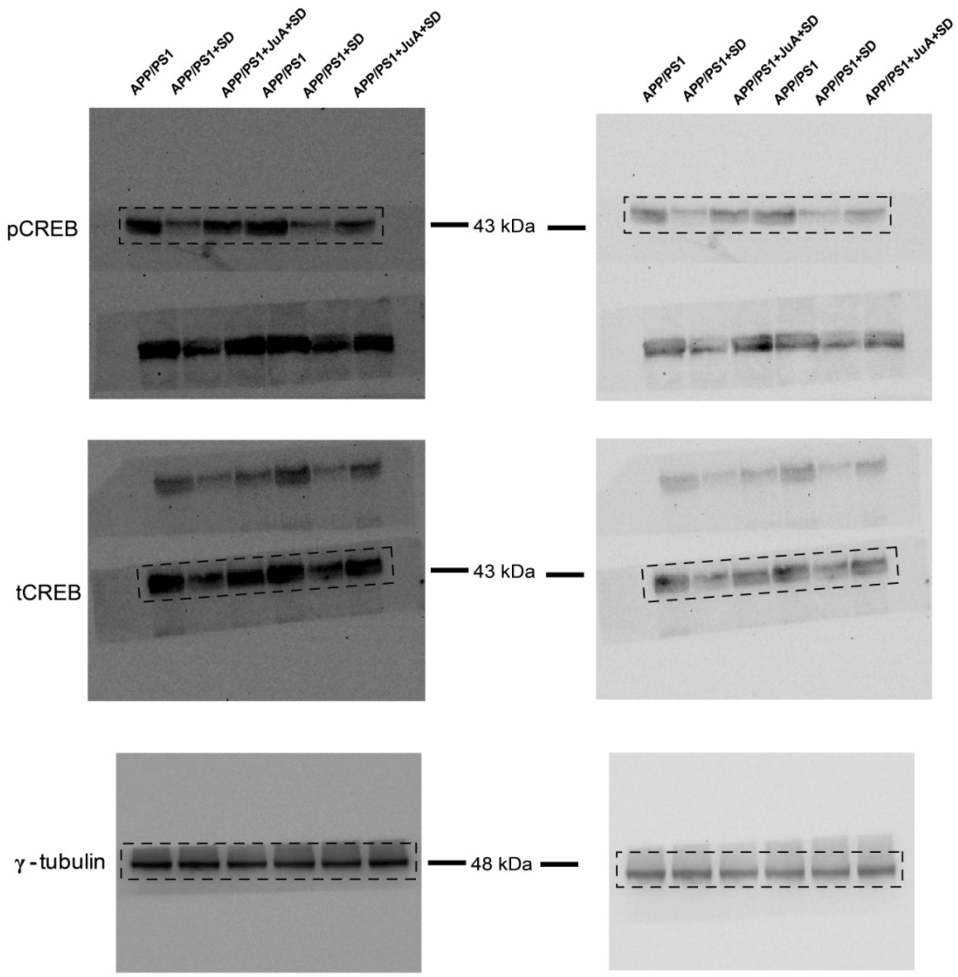

Supplementary figure 6.A (S6.A): Full length images of blots for Fig 5 A in main paper at two different exposures. Dotted rectangles indicate the regions used in the figures. Nitrocellulose membrane was cut before probing with the respective primary antibody.

S6.B

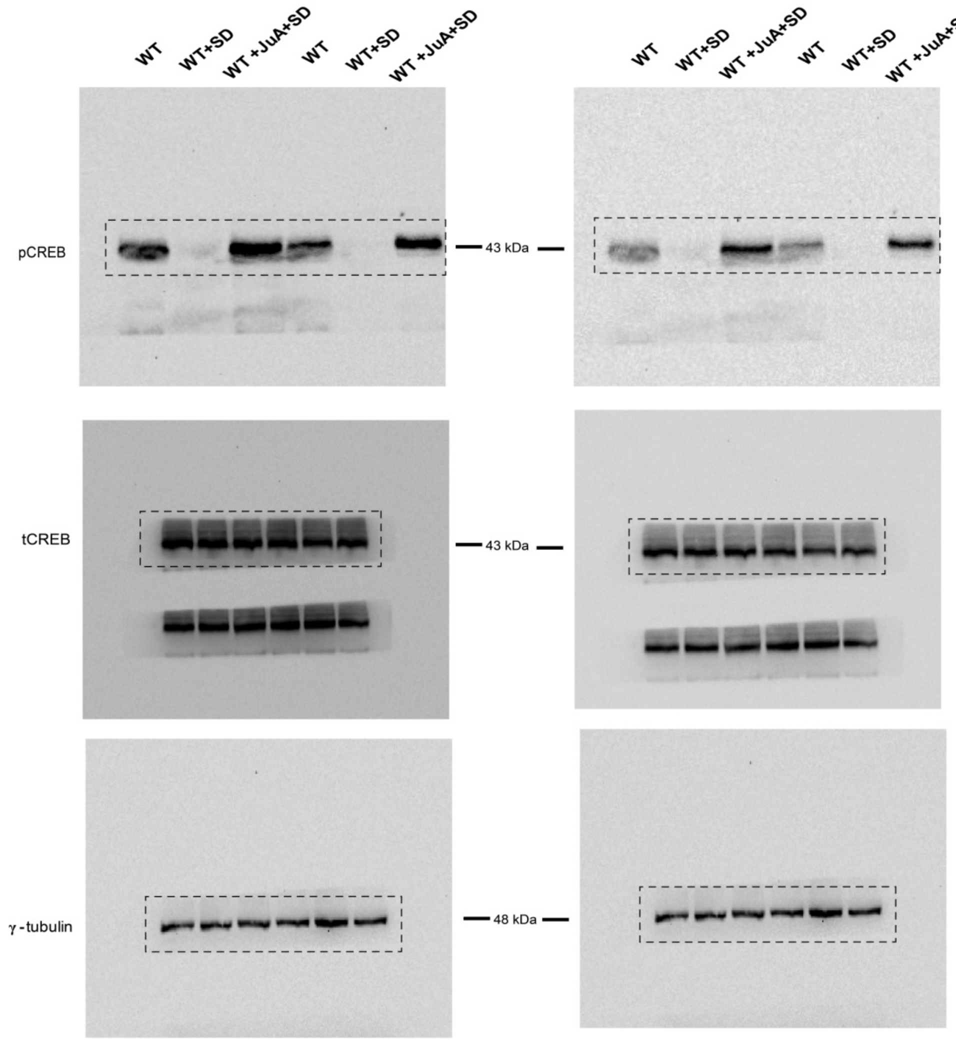

Supplementary figure 6.B (S6.B): Full length images of blots for Fig 5 A in main paper at two different exposures. Dotted rectangles indicate the regions used in the figures. Nitrocellulose membrane was cut before probing with the respective primary antibody.
